# The Role of Trust in Explaining Food Choice: Combining Choice Experiment and Attribute Best–Worst Scaling †

**DOI:** 10.3390/foods9010045

**Published:** 2020-01-03

**Authors:** Ching-Hua Yeh, Monika Hartmann, Nina Langen

**Affiliations:** 1 Department of Agricultural and Food Market Research, Institute for Food and Resource Economics, University of Bonn, 53115 Bonn, Germany; monika.hartmann@ilr.uni-bonn.de; 2 Department of Education for Sustainable Nutrition and Food Science, Institute of Vocational Education and Work Studies, Technical University of Berlin, 10587 Berlin, Germany; nina.langen@tu-berlin.de

**Keywords:** preference, trust, choice experiment, best-worst scaling, latent class analysis, hierarchical Bayesian mixed logit model

## Abstract

This paper presents empirical findings from a combination of two elicitation techniques—discrete choice experiment (DCE) and best–worst scaling (BWS)—to provide information about the role of consumers’ trust in food choice decisions in the case of credence attributes. The analysis was based on a sample of 459 Taiwanese consumers and focuses on red sweet peppers. DCE data were examined using latent class analysis to investigate the importance and the utility different consumer segments attach to the production method, country of origin, and chemical residue testing. The relevance of attitudinal and trust-based items was identified by BWS using a hierarchical Bayesian mixed logit model and was aggregated to five latent components by means of principal component analysis. Applying a multinomial logit model, participants’ latent class membership (obtained from DCE data) was regressed on the identified attitudinal and trust components, as well as demographic information. Results of the DCE latent class analysis for the product attributes show that four segments may be distinguished. Linking the DCE with the attitudinal dimensions reveals that consumers’ attitude and trust significantly explain class membership and therefore, consumers’ preferences for different credence attributes. Based on our results, we derive recommendations for industry and policy.

## 1. Introduction

Over the last decades, food safety and quality have been highly debated and investigated topics in policy, industry, and research. This holds for industrialized as well as emerging countries, such as Taiwan. In Taiwan, food scares and scandals, such as food adulteration [1], food-borne contamination [2], counterfeiting [3], and mislabeling [4], have induced consumer distrust and concerns regarding the quality and safety of food. Additionally, high—and in parts, improper—use of chemical inputs in Taiwanese agriculture [5] has led to illegal levels of chemical residues in food products, with considerable danger for the immediate and long-term health of consumers [6]. For example, in December 2011, the Greenpeace organization (http://www.greenpeace.org/taiwan/zh/publications/reports/food-agriculture/) sampled 58 fresh fruits and vegetables in eight supermarket chains across Taiwan, and detected 36 different pesticide residues above the maximum allowable levels in 43 types of fruits and vegetables. In the same year the Taiwan Food and Drug Administration discovered a major threat to public health caused by phthalate-contaminated foodstuffs sold on the Taiwanese market. This outbreak event is known as the “2011 Taiwan Food Scandal” [7,8]. Most Taiwanese retailers reacted to the numerous food safety incidents by starting in 2011 to display chemical residue test information, particularly for fresh agricultural and food products. Parallel, consumers’ interest in food labels associated with a higher level of product quality and safety has been gaining in relevance [9,10,11].

### 1.1. Thematic Background

There has been considerable interest in studying how consumers, across countries, evaluate and use food quality and safety information [12,13]. Research shows that consumption of organic food has increased [14,15,16], motivated by consumers’ values and health concerns [10,17]. Consumers also associate the origin of foods with product and process quality [9,18]. Previous studies showed that domestically grown food is perceived as fresher and/or of higher quality [19,20]. More generally, research reveals that country (or region) of origin conveys the production country’s (or area’s) reputation for value and quality [19,21]. Food labels can be a source of information for consumers with respect to a product’s quality and safety characteristics. However, the usage of labels for product choice crucially depends on consumers’ perception and trust in the signals [22,23]. Thus, for decision makers in policy and businesses, it is central to understand how consumers perceive and trust food labels, and food product and process characteristics.

Trust is a complex notion, and a multifaceted concept. In the past 30 years, a growing body of literature has emerged across various scientific fields. In the empirical research on food, a number of studies have examined the role of consumer trust in different food institutions [24,25,26,27,28,29,30] for consumers’ perceptions of food risk [26,31,32,33], such as the usage of pesticides [34,35], irradiated foods [36,37,38,39], nanotechnology foods [40,41,42,43], and genetically modified foods [31,44]. Beyond the wide-ranging investigations on consumer trust in risk management settings, there is also an increasing number of works dealing with consumer trust with respect to different typologies of food (see, e.g., [45,46,47,48]) and consumers’ trust in food suppliers and retailers [49,50]. The term “trust” has often been linked to broader categories, such as confidence [26], preference [51,52,53], loyalty [54], risk taking [55], satisfaction [56], cooperation [57], and commitment [58].

According to Mayer et al. [59], trust can be defined as:

… the willingness of a party to be vulnerable to the actions of another party based on the expectation that the other will perform a particular action important to the trustor, irrespective of the ability to monitor or control that other party.

([59], p. 712)

However, it should be noted that despite the extensive research on trust, there is neither consensus among scholars on the definition or conceptualization of trust [60] nor on its dimensions [61]. According to Siegrist et al. [62] and Ding et al. [63], trust can be measured in two dimensions, a specific and a more general manner. Specific trust refers to trust specifically related to the given referent (i.e., trust to the institution or the company) while general trust is presented as trust towards an object or a group entity. Based on the previous literature, consumer trust can also be divided into four conceptual dimensions: (1) Trust belief [64,65,66,67,68]; (2) trust intention [59,64,65,68,69,70,71]; (3) institutional-based trust [59,64,65,68,69,72]; and (4) general trust [28,59,64,65,68,70,73,74,75]. According to Moorman et al. [76], psychological and sociological aspects are the two key components characterizing trust in the marketing area. Taking the purchase of a food product as an example, the former refers to the confidence and belief in the trustworthiness of the related food actors, such as producers, retailers, certification bodies, and labels, and covers the dimension “trust beliefs” [64,65,67,68,70]. “Trust beliefs” stands for the perception of the trustworthiness of a person or object [59,64,65,68]. The latter implies the cognitive, affectional, or behavioral willingness to rely on such actors and relates to the dimension of “trust intention” [64,65,71]. Trust intention is recognized as the intention to engage in trust-related behavior with a specific willingness of the trustor to rely, or intend to rely, on other trustees based on the expectation about the behavior of others though those cannot be controlled [64,65,68,69]. Likewise, “institutional-based trust”, so-called systems trust [70], is considered as an antecedent to trusting intentions and trusting beliefs [59,64,65]. It refers to an individual’s perception that an action is constitutively embedded in an institutional environment that is conducive to a favorable outcome [59,65,68,71]. Thus, an individual intrinsically feels or believes that the macro-level organization or the social environment, in which (s)he performs a transaction, takes on a regulatory role and provides appropriate formal protection [59,64,65,68]. “General trust”, so-called dispositional trust [65,70], is described as the attitude of the general trusting stance and natural tendency of an individual to trust other people or an object; thus, the trustor inherently possesses faith in optimism [64,68,70,71]. It is like a personal conscious choice or strategy to trust others until they prove to be untrustworthy [59,68,70,71]. Consumer trust is a subjective concept and is influenced by an individual’s past experience and perceived reputational value of the object [77].

### 1.2. Methodological Background

Many food-related studies investigate via choice experimental settings the role of labels in consumers’ purchase decisions (see the review by [78,79,80]), or the interaction between food labeling and consumer trust (see the review by [81]). The respective literature so far primarily focuses on western countries with only three studies referring to non-western countries (two peer-reviewed papers, one focusing on Japan ([82] and one on China [83]; as well as a dissertation with data collected in Kenya [84]). Parallel, hybrid choice models (HCMs) have been developed. Those models extend the standard discrete choice experiment (DCE) by explicitly incorporating consumers’ psychological or sociological factors into a random utility framework, thereby better capturing the complexity of the choice process [85,86,87]. HCM covers a number of approaches encompassing latent class [88] or mixed logit models [89] that also include, besides the information obtained from the DCE, e.g., attitudinal factors. Integrated choice and latent variable models [90] also belong to this group. Those models improve the modeling of the decision-making behavior. Our approach goes beyond the HCMs as we combine the findings of two elicitation techniques—DCE and best–worst scaling (BWS)—to provide information about the role of consumers’ trust in food choice decisions in the case of credence attributes in a discriminant manner. Although, Song et al. [91] also combined DCE and BWS, the authors used the identical seven attributes in the DCE and in the BWS settings and analyzed the choice data via HCM, treating each BWS importance score as a single variable. In our study, the attributes in the DCE (four attributes) and the BWS (25 statements) designs are different, with the former referring to characteristics of the product and the latter to 25 attitudinal and trust-based items, which are for the further analysis componentized to five attitudinal and trust dimensions. Thus, in contrast to Song et al. [91] we are able to link consumers’ choice to the attitudinal and trust statements. Furthermore, our approach differs in that we apply DCE using latent class analysis and BWS employing Bayesian estimation. Thus, we use state of the art methods to provide in-depth insights in explaining consumers’ choice for different consumer segments by attitudinal and trust dimensions.

Besides this methodological innovation, our study adds to the literature by revealing in the example of Taiwan—a newly industrialized country—the relevance selected process and food safety standards have for consumers in their purchase decisions, thereby in contrast to previous studies differentiating between consumer segments [92,93] and identifying the role of trust for consumers when buying food products. In summary, our analysis aimed at providing a better understanding of consumer choices, and thus allowing for more meaningful recommendations for marketers and policy makers.

This paper is structured as follows: First, the methods of the discrete choice experiment and best–worst scaling and their application are introduced, followed by a presentation of the empirical results. We conclude with a discussion of our empirical findings, derive practical implications, and suggest directions for future research.

## 2. Materials and Methods

The study combined two elicitation techniques: Discrete choice experiment (DCE) and best–worst scaling (BWS). Both methods are based on random utility theory [94,95].

### 2.1. Data Collection

The questionnaire was formulated in Mandarin and started with two screening questions. To qualify for taking part in the survey, respondents had to be red sweet pepper consumers who were (partly) responsible for the food purchases in their family. Subsequently, participants were asked to complete two stated preference experiments (DCE and BWS as discussed in the next sections). In the last section of the questionnaire, participants were requested to provide information with respect to their food purchase behavior, shopping frequency, and socio-demographic characteristics, such as gender, age, and income. The questionnaire was tested to ensure the comprehension of the questionnaire. The survey was conducted by five trained interviewers in front of supermarkets (the two hyper-supermarkets Taisuco (http://www.tsctaisuco.com.tw/) and Carrefour (http://www.carrefour.com.tw/) were chosen based on the convenience of the location, the customer flow, and the amount of fresh agri-food product categories) in the three largest cities (New Taipei, Kaohsiung, and Taichung) of Taiwan in the form of computer-assisted web interviews. The majority of the respondents completed the survey within approximately 15 min. To reduce possible self-selection bias, we trained our interviewers to actively encounter randomly every second person leaving the checkout counter of the supermarkets. This was done to ensure that, e.g., not only young or female consumers were approached. Combining computer-assisted personal interviews with traditional web survey techniques allowed us to overcome the problem of coverage error linked to the latter [96] and non-response error linked to the former [97].

### 2.2. Discrete Choice Experiment (DCE)

Discrete choice experiments are based on Lancaster’s new demand theory [95,98], which assumes that consumers’ derive utility from a variety of product characteristics. Participants are presented with multiple choice sets and asked to choose the product among a given choice set of alternatives that holds the combination of attributes that maximizes his/her utility [99,100].

Our DCE was conducted to investigate consumers’ food preference and heterogeneity regarding different food quality and food safety information. In this study, fresh unpackaged red sweet peppers were selected as the study object. This product seemed especially suitable for our analysis because first, it is part of Taiwanese people’s daily diet [101,102], and second, it is one of the few fresh agri-food products permitted to be imported into Taiwan from mainland China. In fact, red sweet peppers are available on the Taiwanese domestic market in conventional and organic quality from the three countries considered in the study: Taiwan, China, and Japan. Country of origin (COO) and production methods (organic and conventional) are both important attributes influencing perceived quality and trust in a product’s overall quality [103,104]. Besides these two attributes, we considered price and chemical residue testing information (see Table 1) as characteristics in the DCE. Those four attributes were identified as the most relevant selection criteria for consumers in their purchase of red sweet peppers based on two focus group discussions held in March 2014 by the first author of this paper via video meetings with Taiwanese consumers primarily responsible for their household food purchase (*n* = 17). Those food market experts were recruited via social media networks. Out of 12 potential attributes extracted from previous literature (COO, easiness in preparation; chemical residue testing; visual appearance of the product; production methods, e.g., organic certification; product’s shelf life; taste of the product; health claim; word of mouth information; seasonal product; and sense of touch) participants were asked to select those five attributes most important to them when buying fresh fruits and vegetables. In addition, participants were encouraged to express their opinions and reasons about why they choose the respective attributes.

**Figure 1:**
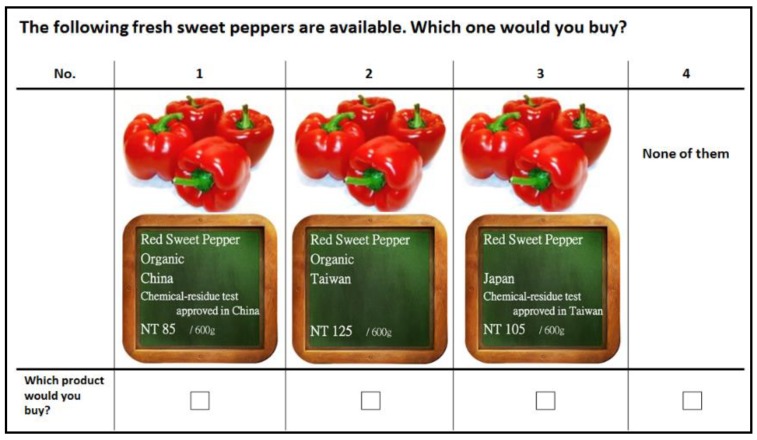
A choice task example in DCE (English translation).

Using NGENE version 1.1 [105], we identified an efficient unlabeled choice design with a D-error value of 0.237, which is smaller than the D-errors of other design alternatives, such as the sequential orthogonal design (0.420) and the least efficient simultaneous orthogonal design (2.018) [105]. The D-error is the most common criterion for evaluating the efficiency of experimental choice designs [106]. A design with the lowest D-error measure is a D-efficient design. Efficient designs can be generated using two different approaches. The first approach assumes that prior parameters are known with certainty by the researchers (e.g., [107,108]), whereas the second one uses prior parameter distributions (Bayesian efficient design) [109]. We used the latter approach. For that, a pilot study was carried out in April 2014 with Taiwanese consumers (*n* = 290) from a convenience sample using an internet-based choice experiment questionnaire in Taiwan. The pilot study’s parameter estimates were used as the prior information to derive the asymptotic variance-covariance matrix and subsequently the D-efficient design. Based on an iterative process, the most efficient design with the smallest D-error was derived. The final design consisted of 36 choice sets. However, as 36 choice tasks would lead to respondents’ fatigue, the choices were allocated in six blocks by NGENE software [105] via the design generation process, each consisting of six choice situations. Respondents were randomly assigned to the six blocks. In each choice task, consumers were asked to make a choice between three red sweet peppers that varied in the levels of the four product attributes presented in Table 1. Though, all attributes depicted on the products in the choice tasks exist in the market, this does not hold for all combinations of attribute levels (e.g., the link between the chemical residue test and the country where it is approved). We also provided participants with an “opt-out” option, which ensured that participants were not forced to choose a product they normally would not purchase. In order to make the choice experiment as tangible as possible, the attribute levels were visualized using pictures and text as shown in Figure 1 (translated version).

We applied standard latent class analysis (LCA) [110,111] to the choice experimental data to identify different consumer segments. Latent class choice models assume that respondents can be categorized into two or more classes sharing unobserved characteristics that affect choice, in our case the choice of red sweet peppers, differentiated by different attribute levels. LCA allowed simultaneous determination of both the consumer’s product choice and group membership, thereby segmenting the sample into internally homogenous subgroups regarding their preferences [110,111,112,113,114,115].

Following the random utility theory [95], the utility of individual n (n=1,…,N) from choosing alternative *j* (j=1,…,J) is the sum of a systematic observed component ($\beta_{n}X_{nj}^{\prime }$) and a random error term ($\varepsilon_{nj}$) [110]:

(1) $$U_{nj}=\beta_{n}X_{nj}^{\prime }+\varepsilon_{nj},\;\beta_{n}\sim N\left(\alpha ,\;D\right).$$

In the LCA model, the utility of alternative j∈J to individual n, who belongs to a specific class, s, can be expressed as [112,113]:

(2) $$U_{nj|s}=\beta_{s}X_{nj}^{\prime }+\epsilon_{nj},$$

where the $X_{nj}^{\prime }$ is a vector of explanatory variables associated with alternative j and individual n, $\beta_{s}$ is a class-specific parameter vector associated with the vector of explanatory attribute variables and $\epsilon_{nj}$ is the error term. The probability of individual n choosing alternative j from a particular choice set of alternative *J* is conditional to the fitting of the individual to class s, which can be estimated using Equation (3) [110]:

(3) $$P_{nj|s}=\frac{\exp\left(\beta_{s}X_{nj}^{\prime }\right)}{\sum_{j=1}^{J}\exp\left(\beta_{s}X_{nj}^{\prime }\right)}.$$

In addition, the belonging of individuals to the S classes is determined by a classification model as a function of individual specific invariant characteristics. For each respondent, the class member probability ($C_{ns}$) of individual n belonging to class s can be computed as Equation (4):

(4) $$C_{ns}=\frac{\exp\left(\gamma_{s}Z_{n}\right)}{\sum_{s=1}^{S}\exp(\gamma_{s}Z_{n})},$$

where $\gamma_{s}$ is the class membership vector estimates and $Z_{n}$ is a vector of individual specific invariant variables that enter the model for class membership. According to Boxall and Adamowicz [112], a vector-labelled $Z_{n}$ as covariates can be used as a proxy for individual motivating factors influencing the choice. This vector, Z, consists of both the observable indicators of the latent attitudes expressed by the respondent and of the observable socioeconomic characteristics, such as gender. The N individuals can be divided into S latent classes. Preferences are assumed to differ between latent classes but to be homogeneous within classes. The joint probability that individual n belongs to a specific class, s, and selects alternative j can be shown by Equation (5):

(5) $$P_{nj}=\sum_{s}^{S}P_{nj|c}C_{ns}=\sum_{s=1}^{S}\frac{\exp\left(\beta_{s}X_{nj}^{\prime }\right)}{\sum \exp\left(\beta_{s}X_{nj}^{\prime }\right)}\times \frac{\exp\left(\gamma_{s}Z_{n}\right)}{\sum \exp\left(\gamma_{s}Z_{n}\right)},$$

where $\beta_{s}$ is the parameter vector of individuals in the class s, and $\frac{\exp\left(\gamma_{s}Z_{n}\right)}{\sum \exp\left(\gamma_{s}Z_{n}\right)}$ is the probability of the individual n falling into latent class s. The number of latent classes is determined by the researcher based on the statistical measures of fit, such as the Bayesian information criterion (BIC) and Akaike information criterion (AIC).

In addition, we calculated the segment-specific willingness-to-pay (WTP) values for each attribute level for the detected consumer segments by dividing the attribute level coefficient by the price coefficient. Due to the use of effect coding in the choice model, we calculated the mean WTP according to: $\mathrm{WTP}=-\frac{2\beta_{\mathrm{attribute}\;\mathrm{level}}}{\beta_{\mathrm{Price}}}$ [116,117,118]. We used the delta method introduced by [119] to generate the 95% confidence intervals for the WTP estimates.

### 2.3. Best–Worst Scaling (BWS)

Although the DCE method allows the combination of a product’s attributes and levels to examine consumer preference with respect to a specific food product, it does not provide information on an individual’s attitudinal and trust perceptions driving those choices. Therefore, many studies include rating scales (e.g., Likert measure points) to obtain information on consumers’ attitudinal and trust perception. While rating tasks are easy for respondents to answer, they may ineffectually discriminate between rating statements [120], as respondents are not forced to make a choice between items, allowing them to rate multiple items as being of equally high importance. In addition, it is difficult to interpret what the rating scale values actually mean [121]. To overcome these weaknesses, we employed the best–worst scaling method [122] to uncover the attitudinal and trust factors underlying consumers’ food choices on a reliable basis. Best–worst scaling (BWS), also known as maximum difference scaling, is an annotation scheme that exploits this comparative approach [123,124]. In BWS experiments, respondents are asked to choose the best and worst option among a number of statements. In this study, we used the so called ‘object case’ of BWS [122]. Thereby, respondents are forced to consider trade-offs and discriminate between options as in real life [125]. Choice frequencies are the metric that allow reveal information on the order and strength of the importance of all objects to be revealed. The method was introduced by [126] and first applied in the study of [122].

The BWS experiment covered nine attitudinal dimensions with a total of 25 statements related to attitudinal and trust factors. These were derived from the literature as well as our own consideration and were adapted to the context of the study (see Table 2). The balanced incomplete block design (BIBD) [127] has been frequently used in the BWS setting; however, as a BIBD is subject to the symmetry condition, the number of possible BIBDs is limited. In the present study, an orthogonal frequency balanced design using MaxDiff Designer v.6 [128] was generated to maximize the BWS design efficiency [129,130]. Orthogonality ensures that differences among items varied independently over choice sets while balance confirms that all items appeared with (nearly) equal frequency in the BWS questionnaire. Given a 25-statement BWS questionnaire, 300 BWS choice tasks were generated to control for context effects that may alter respondent choice processes [128]. To avert respondent fatigue, the choice tasks were divided into 30 blocks, where each version had 10 choice sets displaying 5 statements at a time. The generated BWS choice tasks satisfy the optimal design characteristics in terms of perfectly balanced frequency (on average, each BWS statement is displayed across all 30 blocking versions of the BWS questionnaire 60 times), orthogonality, positional balance (on average each statement appears 12 times at the same position (S.D. = 0.522)), and connectivity among tasks (all statements are linked directly) (see Appendix A) [128]. After ensuring a balanced and nearly orthogonal BWS design, the BWS situations were randomized and each respondent was randomly assigned to a BWS block while orthogonal properties hold after the randomization blocking process. Each BWS blocking version has the same sample size to maintain its statistical properties.

**Figure 2:**
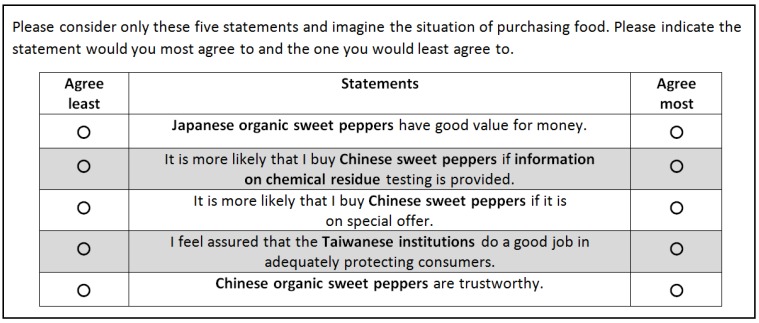
A choice task example of BWS (English translation).

In addition, each statement appears equally often on each of the five positions within the BWS sets to prevent any position bias [131]. In each BWS task, respondents were asked to choose the statements that most and least represent their attitude when purchasing food (mix of questions regarding food purchases in general and red sweet peppers in particular) (see Figure 2).

First, hierarchical Bayesian estimation of the mixed logit model was applied to analyze the BWS choice data. We started with the general formula of random utility theory [95]:

(6) $$U_{nj}=\beta_{n}X_{nj}^{\prime }+\varepsilon_{nj},$$

where $U_{nj}$ is the utility obtained by an individual n choosing item j (j=1,2,…,J). $\beta_{n}$ is the individual-specific preference parameter vector, $X_{nj}^{\prime }$ is the vector of observable explanatory variables including the chosen alternative j, and $\varepsilon_{nj}$ is the stochastic error term. In the BWS dataset, the most important item is coded as 1, where the least important item is coded as −1, and the non-chosen items are coded as 0.

It is assumed that an individual, n, chooses item j and $j^{\prime }$ as the most important and the least important item in a choice set, respectively, out of a choice set of *J* items. Thus, the utility difference between $U_{njt}$ and $U_{nj^{\prime }t}$ is then greater than all other J(J−1) possible differences among the other items in the choice set. Namely, in our case, each choice scenario of 5 items would have 5(5−1)=20 possible best–worst combinations a person could choose. Following Louviere et al. [124], the choice probability of the individual n of choosing item j as the best and $j^{\prime }$ as the worst can be written as:

(7) $$P=\frac{\exp\left(\beta_{n}X_{nj}^{\prime }-\beta_{n}X_{n\;j^{\prime }}^{\prime }\right)}{\sum_{\begin{array}{c} j,\;j^{\prime }\in J\\ j\neq \;j^{\prime } \end{array}}^{}\exp\left(\beta_{n}X_{nj}^{\prime }-\beta_{n}X_{n\;j^{\prime }}^{\prime }\right)}.$$

In hierarchical Bayesian estimation, based on Equation (6), we estimated the parameters at the individual level and the coefficient vector, $\beta_{n}$, for the individual, n, can be expressed by dividing it into an individual characteristic vector ($Z_{n}$) and the parameter matrix, Γ, shown in Equation (8):

(8) $$\beta_{n}=\Gamma Z_{n}+\delta_{n},\;\delta_{n}\sim N\left(0,D\right),$$

where $\delta_{n}$ is the error term assuming that a normal distribution with a mean of 0, and D represents the covariance between the partworth estimated values [135]. During the analytical process, a parameter’s posterior distribution is computed by combining its prior distribution for each partworth estimate with the likelihood determined based on the choice data. Thus, Equation (9) plays a role of identifying the prior distribution for the prior distributions of Γ (follows a normal distribution) and D (follows an inverse-Wishart distribution) that were established to complete the hierarchical Bayesian modeling procedure [135]:

(9) $$\begin{array}{c} \Gamma \sim N\left(a,\;A\right)\\ D\sim IW\left(w,\;W\right). \end{array}$$

Along with the above procedures and assumptions, choice data were drawn from a conditional distribution by generating the hierarchical structure shown in Equation (10):

(10) $$\begin{array}{c} \Gamma |\;D,\beta_{n}\\ D|\beta_{n},\;\Gamma \\ \beta_{n}|\Gamma ,D. \end{array}$$

When the covariance matrix (D) and the individual level value of an attribute ($\beta_{n}$) are given, the coefficient estimates (Γ) for the variable of an individual characteristics ($Z_{n}$) can be extracted. Therefore, we were also able to extract the D when $\beta_{n}$ and Γ were obtained, as well as $\beta_{n}$ when Γ and D were gained. This process repeats iteratively until a parameter value converges to draw a distribution of the individual parameters [135]. As a result, we obtained individual attitudinal importance scores for each of the BWS statements. Compared to the multinomial logit model or the mixed logit model with classical maximum likelihood estimation, the hierarchical Bayesian estimation allows for more precise estimates of individual-level partworth values [136,137] by combining information on the distribution of utility values across the entire sample with the specific choices of the individual [110,138].

Second, following the analytic methodology of [139], a principal component analysis (PCA) with oblimin rotation was used to identify latent constructs as the drivers of food purchasing decisions behind the 25 attitudinal items [140,141]. The number of components in the PCA was determined by the scree test, and the parallel analysis method [142,143,144,145] using STATA version 15. The former identifies the optimal number of components to retain by examining the scree plot of the eigenvalues and looking for the “elbow” point in the data where the curve flattens out. The latter compares the components’ eigenvalues derived from the own data set with those of a large number of data matrices obtained from random values of data with the same dimensionality (same number of observations and same number of variables). As long as the estimated eigenvalues from the own data exceeds the mean of the eigenvalues of the random data the component is retained. The parallel analysis is considered to be among the most recommended factor/component retention methods [143,144,145,146] for assessment of the dimensionality of a variable set. Afterward, for each component the individual-level important scores were obtained from the respective values of the underlying BWS items.

### 2.4. Combining DCE and BWS

**Figure 3:**
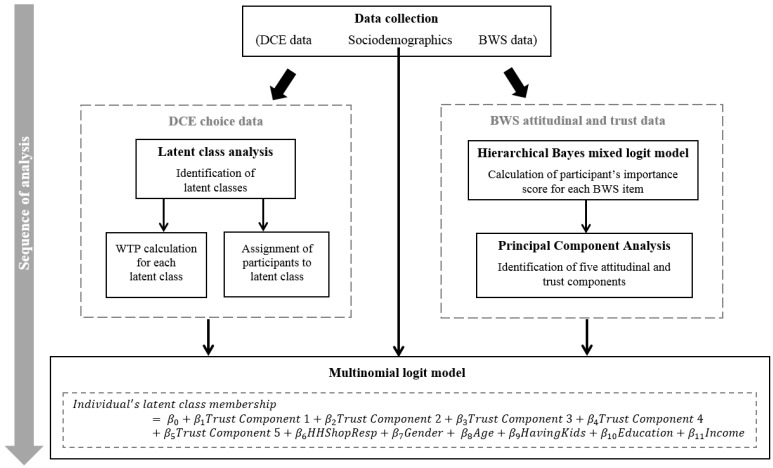
Research flow diagram (stepwise analysis (in the (long-format) dataset, as each individual contributed to 24 rows in the DCE data and 100 rows in the BWS data, the DCE and BWS analysis were not estimated simultaneously but sequentially. This is due to the reason that none of the exiting software, at current, is capable of estimating two different choice datasets concurrently in a model)).

In order to better understand class membership as detected in the latent class analysis, we followed the approach of [112]. Thus, by estimating a multinomial logit model with each participant’s latent class membership as the dependent variable and attitudinal as well as trust factors (from the BWS and PCA analysis) and sociodemographic characteristics as independent variables, we attempted to explain class membership. Figure 3 provides an illustration of the analysis flow.

## 3. Results

### 3.1. Sample Demographics

In total, 790 people joined the survey. Excluding those not responsible for their household’s food shopping and/or not consuming red sweet pepper resulted in 459 (58% of all participants) valid responses. Thus, we far exceeded the target sample size (*N* = 237). The latter was determined using a power analysis assuming three alternatives for each choice task, a 5% margin of error, and a desired 95% confidence interval. Our target sample size is consistent with guidelines in the conjoint analysis methodology [147]. Of the 459 respondents, 72.1% were female, 61.9% were married, 33.3% had children under the age of 18 living in their household, and approximately 50.8% had completed university or higher education, and 39.0% stated that their household average monthly net income was below NT 60,000 (approximately US$2001) (and 45.3% above NT 60,000). The respondents’ average age was 39.2 years. Compared to the Taiwanese population, the sample is biased towards younger and higher-educated segments. The high proportion of females in the sample is desirable because they are the primary food shoppers in most Taiwanese households [148]. Summary statistics for the demographic variables are presented in Table 3.

### 3.2. Identifying Consumer Segments Based on DCE Data

Determining the optimal numbers of classes required a balanced assessment of the five major criteria reported in Table 4 [149]: Log-likelihood, percent certainty (Pct. Cert.) [150,151], consistent Akaike information criterion (CAIC) [152], Chi-square, and Bayesian information criterion (BIC) [153]. For this study, all indicators improved as more segments were added, supporting the presence of multiple segments in the sample. The four-segment solution (Table 4) provided the best fit to the data. Although indicators further improved as more segments were added, the changes were much smaller from a four- to a five-segment model compared to the move from a three- to a four-segment model. Furthermore, the model interpretability is as important as the statistical tests [113] and was best for the four-class model.

Table 5 summarizes the results of the four-class model and provides information on the attribute importance scores as well as on the partworth utilities (with respect to the attribute price information provided on the coefficient) and the corresponding standard errors for each attribute level for the four different consumer segments identified in the latent class analysis. To allow for comparability between classes, attribute importance scores were standardized to sum up to 100% across all attributes of each segment. For the same reason, partworth utilities were re-scaled and zero-centered. Positive (negative) partworth values indicate an increase (decrease) in utility relative to the average level of the respective attribute.

As shown in Table 5 attribute importance considerably differs between the four classes. For segments 1 (*Japan Lovers*) and 2 (*Domestic Supporters*), COO is by far the most important attribute (attribute importance: 59.8% and 71.7%, respectively). Consumers of both segments strongly dislike China as a COO. Price is the second most important attribute in both segments (attribute importance: 24.5% and 16.2%, respectively) followed by chemical residue testing (CRT) information (attribute importance: 8.9% and 6.9%), with production methods (attribute importance: 6.8% and 5.2%) being least important. As expected, *Japan Lovers* and *Domestic Supporters*, like the other groups, reveal a negative price elasticity and prefer organic to conventional red sweet peppers. Regarding the attribute levels for CRT information preferences, both groups prefer CRT information to no information. However, while *Japan Lovers* obtain an above-average utility from CRT approved in either the production country or Taiwan—though with a higher preference for the former—*Domestic Supporters* highly value CRT approved in Taiwan.

In contrast to segments 1 and 2, for respondents in segment 4 (*Process Quality Supporters*), the production method, COO, and CRT information is of similar importance (attribute importance: 32.5%, 32.3%, and 28.0%, respectively) while price plays a minor role in consumers’ purchase decisions of red sweet peppers (attribute importance: 7.3%). *Process Quality Supporters* prefer Taiwanese over Japanese foods and dislike products from China. They prefer organic products and those which are CRT approved in Taiwan. Finally, for consumers of class 3 (*Price Conscious Consumers*), price is by far the most important attribute (attribute importance: 54.0%). COO takes second place, but with an attribute importance score of 27.4%, it is of much lower relevance. *Price Conscious Consumers* especially prefer Japanese products but also like those from Taiwan while products originating from China are disliked, though to a lesser extent compared to the other three segments. CRT information is the third important attribute (attribute importance: 15.3%), with CRT approved in Taiwan being preferred. No CRT is less disliked than CRT approved in the production country. The production method is of little relevance (attribute importance: 3.4%) for consumers of this group. For members of the *Price Conscious Consumers*, the no-choice option is linked to a high negative value (−320.63), implying that not purchasing any red sweet pepper is associated with a high utility loss. Accordingly, the opt-out option is hardly chosen (0.17% for the share of the opt-out decision). In comparison, the share of deciding for the no-choice option is 1.24%, 8.69%, and 13.87% for the *Process Quality Supporters*, *Japan Lovers*, and *Domestic Supporters*, respectively, which is 7 to 82 times higher.

To ease the comparison of the attribute-level importance between attributes of one consumer segment as well as between segments, WTP measures were calculated (see Table 6). Table 6 reveals that *Domestic Supporters* have a high WTP of NT 13.07 (approximately US$0.44) per 600 g for fresh red sweet peppers originating from Taiwan while they would buy red sweet peppers originating from China only at a high discount (NT −13.46) (approximately US$−0.45). All other attribute levels just marginally influence *Domestic Supporters*’ WTP. The latter also holds for consumers belonging to the *Japan Lovers* class. This group is, however, willing to pay extra for products originating from Taiwan (NT 3.94) (approximately US$0.13), and even more for those from Japan (NT 5.36) (approximately US$0.18). In line with the *Domestic Supporters*, they would only buy Chinese red sweet peppers at a high discount (NT −9.30) (approximately. US$−0.31). Furthermore, *Process Quality Supporters* exhibit a high WTP for several attribute levels, e.g., organic products (NT 13.31) (approximately US$0.44) and Taiwan-authorized chemical residue testing information (NT 10.90) (approximately US$0.36) while they only would buy products from China (NT −15.35) (approximately US$−0.51), conventional products (NT −13.31) (approximately US$−0.44), or those with no CRT information (NT −12.05) (approx. US$−0.40) if they obtain a high discount. Finally, the *Price Conscious Consumers* segment is characterized by very low WTP values for every attribute level.

### 3.3. Identifying Consumers’ Attitude and Trust Based on BWS Data

Consumers’ attitude and trust as obtained from the BWS data were estimated through hierarchical Bayesian analysis. Information on the importance consumers attach to each of the 25 attitudinal and trust statements is provided in Appendix B. Appendix B reveals that respondents’ trust with respect to products originating from China as well as trust in Chinese institutions is very low. All attitudinal and trust statements with reference to China (statements 2, 5, 8, 11, 18, 21, and 24) have an average importance score below one (between 0.05 and 0.75). In contrast, the average importance scores for all other statements range between 1.98 (I generally like to consume conventional red sweet peppers produced in Japan) to 8.42 (It is more likely that I buy Taiwanese red sweet peppers if they have information on chemical residue testing).

**Figure 4:**
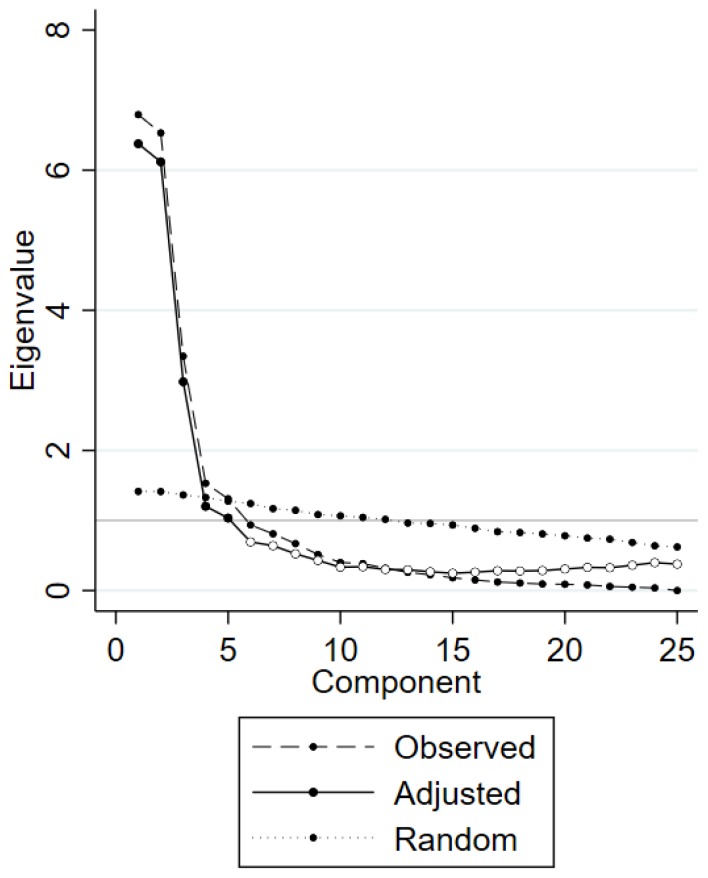
Diagnostic scree plotting graph (performed by parallel analysis) for principal component analysis (PCA) on estimated BWS importance scores data.

Following the analytic method presented in the paper of [139], we performed principal component analysis (PCA) based on the individual-level importance scores obtained from the hierarchical Bayesian estimation to check the dimensionality of the BWS data. Examining the eigenvalues (Table 7) and scree plot (Figure 4) with a permutation test approach and applying oblimin rotation (see Table 8), we obtained a factor solution containing five components. Factor loading of all attitudinal statements exceeded the cut-off value of 0.4. A cut-off for the statistical significance of the factor loadings of 0.40 was used, according to the guidelines presented by Hair et al. [154] for sample sizes greater than 200. A more conservative cutoff value would have been 0.50. The reasons for deciding on this lower value were first, that this was an exploratory factor analysis, and the factors detected were reasonable. Second, with a sample size of 459, it seems unlikely that the weaker factor we found (“I feel assured that the Chinese institutions do a good job in adequately protecting consumers” with a factor loading of 0.454) was random noise. Together, the five components explained 78.03% of the total variance. Table 9 shows the initial eigenvalues, the variance explained by each component and the cumulative variance.

The first component includes five items related to the trust and liking of organic products from Japan and skepticism toward Taiwanese conventional products (trust in Japan), while the 10 items under the second component refer to the trust and liking of organic and of Taiwanese products and institutions with a skepticism towards Japan (trust in Taiwan and organic). The third component covers three items referring to trust in Chinese products (trust in Chinese products). The two items of the fourth component include variables relating to a lack of trust in the superiority of organic products (no trust in organic). Finally, four of the five items of the last component refer directly to the trust and liking of organic products from China as well as the trust in Chinese institutions (trust in Chinese organic products). For the fifth item (‘It is more likely that I buy Taiwanese red sweet peppers if they have information on chemical residue testing’), this is not the case.

### 3.4. Characterizing Consumer Segments with Respect to Attitudes and Trust

To identify the relevance of attitudes and trust in determining differences between segments in the choice model (see Section 3.2), a latent segmentation model was estimated. For each individual in the sample, factor scores were calculated for all five attitudinal and trust components identified in Section 3.3. Subsequently, we estimated two multinomial logit models taking each participant’s latent class membership as the dependent variable and the individual attitudinal and trust factor scores alone (Model 1) and in combination with sociodemographic information (Model 2) as independent variables. Both models fit the data well according to the likelihood ratio (LR) Chi-square test (see Table 10). Pseudo R-square measures indicate that the models explain 23% and 25% of the variance, respectively. Hence, the trust-related components alone explain 23% of the variance while sociodemographics only add two percentage points to the model fit.

The segment membership parameters are summarized in Table 10. The parameters of the class *Japan Lovers* were normalized to zero. This is necessary to allow for identification of class membership of the three other segments. It, however, implies that the results of the segments *Domestic Supporters*, *Price Conscious Consumers*, and *Process Quality Supporters* have to be interpreted relative to the segment of *Japan Lovers*.

As expected, attitude towards and trust in products, labels, origins, and institutions have a considerable influence on segment membership. Table 10 indicates that all components but ‘no trust in organic’ significantly influence class membership with respect to the purchase of red sweet pepper. Not surprisingly, high ‘trust in Japanese organic products’ reduces the likelihood of a consumer to belong into any other segment but *Japan Lovers*. Also, in line with expectations, high ‘trust in Taiwan and organic’ increases the likelihood to be part of the segments *Domestic Supporters* and *Process Quality Supporters* relative to the segment *Japan Lovers*, respectively. Those consumers with high ‘trust in Chinese products’ or ‘high trust in Chinese organic products’ have a greater likelihood to be a member of the *Price Conscious Consumers* or the *Process Quality Supporters* than of the *Japan Lovers*. The influence of the trust components on class membership holds for Model 1 as well as for Model 2. The latter model also investigates the influence of sociodemographic factors on classification. The findings indicate that compared to *Japan Lovers*, consumers belonging to the segment of *Domestic* *Supporters* are more likely to have full shopping responsibility for their household and to have a lower level of education. *Price Conscious Consumers* have, compared to *Japan Lovers*, a higher probability to be female and to have no kids. Also, this group has a lower probability to have an above-college educational level when compared to the reference segment. Comparing *Process Quality Supporters* and *Japan Lovers*, it becomes obvious that sociodemographics have no influence on class membership.

## 4. Discussion

The present study is the first that combines a latent class model for discrete choices with BWS to uncover attitudinal and trust dimensions. This approach allows us (1) to distinguish consumer segments that differ in their preferences for selected process and food safety standards, and (2) to reveal the importance of consumers’ attitudes and trust in explaining consumers’ group membership. Our study illustrates that combining DCE and BWS allows for a better understanding of the drivers of consumers’ food choices and thus provides additional important information for decision makers in the economic and policy arena.

Based on our analysis, we could distinguish four segments of Taiwanese consumers based on differences they attach to various process attributes and price. The largest group can be described as *Japan Lovers*. According to Huang [155], the trend of Taiwanese consumers to buy Japanese products can be explained by the positive cultural image of “Japanese-ness”. In our analysis, we showed that respondents belonging to the segment *Japan Lovers* reveal a higher level of trust in Japan relative to the other three consumer segments identified. Furthermore, Ma et al. [156] showed that Japanese products have established a reputation of being of high quality. Quality seems an important driver for this consumer segment’s purchase decisions as this group is not interested in special offers and perceives the generally more expensive Japanese products [157] as better value for the money (see Table 9 and Table 10).

The second largest group (26.1%) identified in our study, *Domestic Supporters*, has been detected in previous DCE-based research investigating the relevance of COO in consumers’ food purchase decisions, however, with slightly smaller segments compared to our findings [103,158]. Our segment of *Domestic Supporters* has a high WTP for products originating from Taiwan. According to our results, this originates from a high level of institutional-based trust in the Taiwanese government and its regulatory and controlling power as well as a high level of trust belief in the quality of Taiwanese products. *Domestic Supporters* have a tendency towards ethnocentrism [156]. As revealed in previous research, consumers with a higher level of ethnocentrism favor domestic to foreign goods, perceive the quality of domestic food products higher than those of foreign goods, and have a higher purchasing preference for the former [19,159]. In line with [158], less-educated consumers were found to be more likely in the segment of *Domestic Supporters*.

For the *Price Conscious Consumers* identified in our study, process attributes are of relatively low relevance when buying red sweet peppers. The production method does not impact, and COO and CRT have only a small impact on consumers’ WTP in this group. Price is the determinant that primarily drives consumers’ purchase decisions in this segment (attribute importance 53.95%). *Price Conscious Consumer* segments have also been found in previous DCE-based studies investigating the relevance of process characteristics in consumers’ food purchase decisions, with some studies identifying a segment of a similar size (around 20%, e.g., [160,161,162]) while others reveal a considerably larger group of *Price Conscious Consumers* [17,118,163,164]. The class determinant estimates revealed that male respondents were more likely to be in the class of *Price Conscious Consumers*, a result in line with the findings of [160,164]. In addition, our results indicate that respondents in this segment are less educated and more likely to not have children.

As indicated above, members of the price-conscious segment attach a higher importance to chemical residue test information compared to the production method. Considering that organic certification implies the logic of zero chemicals, this either reflects that respondents are not aware of the core standards behind the organic label and/or do not trust organic labels. According to Tung et al. [14] and Chen [165], Taiwanese consumers indeed have some doubt that products labeled as organic are always grown without using synthetic fertilizers, pesticides, and chemicals. Based on a literature review, Yiridoe et al. [166] came to the conclusion that consumers have difficulties in understanding the complexity of organic standards. Chemical residue test information is easier to understand when compared to organic standards, which might be of special relevance for the price-conscious segment as it consists of a higher share of less-educated consumers.

The analysis provides additional interesting insights regarding this group of consumers. *Price Conscious Consumers* are characterized by a relatively low trust in Japan and a relatively high trust in Chinese products in general, and in Chinese organic products more specifically (see Table 10). This holds despite the fact that this consumer segment also has the lowest WTP for Chinese products (see Table 6). Festinger’s theory of cognitive dissonance might provide an explanation for this finding [167]. With Chinese products typically the cheaper alternative, and given the dominant relevance of price for members of this segment, *Price Conscious Consumers* will often end up with Chinese products in their shopping cart. A lack of trust in and liking of Chinese products and a high trust and liking of Japanese and Taiwanese products would lead to dissonance with the choice behavior. The stronger the latter, the higher the attractiveness of the unchosen alternative compared to the actual choice. One way for consumers to relax or correct this disturbing and unpleasant psychological state is a dissonance-related attitude change [168], implying a correction towards a more positive perception of Chinese products and a higher trust in China.

For the segment of *Process Quality Supporters* with a share of 20.8%, only slightly smaller than the group of *Price Conscious Consumers*, but in contrast to the latter, price is of little importance for the former. This group attaches a much higher importance to the attributes, production method and CRT information, than any other segment. Members of this group are willing to pay high price premia for organically produced red sweet peppers as well as for those with a CRT approved in Taiwan, indicating that for members of this segment, health and food safety are of high importance. This assumption is supported by [165], who found that health concerns are the most important determinant for Taiwanese consumers forming a positive attitude towards organic. Along the same line, Hasimu et al. [169] and Xie et al. [170] showed that health and food safety are the core motives for consumers in China to buy organic foods. Our analysis reveals that a high level of trust (component 1, 2, 3, and 4 in Table 10) increases the probability of a consumer belonging to the segment of *Process Quality Supporters*. This result is in line with [171], who showed that trust in labeling is essential for Taiwanese consumers’ intent to purchase organic products.

The four segments identified reveal the difference in importance Taiwanese consumer groups attach to different attributes when buying red sweet peppers. From a marketing perspective, this implies that a ‘one size fits all’ marketing strategy is inappropriate. Thus, marketers need to develop segment-specific offerings in order to better target the needs of their customers. For the two largest segments, the *Japan Lovers* and *Domestic Supporters*, by far the most important attribute is COO, though with a clearly distinct preference for the attribute levels. In addition, both groups reveal very high opt-out ratios (*Japan Lovers*, 8.7%; *Domestic Supporters*, 13.9%). Thus, to secure the loyalty of customers and to extract a price premium in both groups, retailers need to have red sweet peppers from Japan as well as Taiwan in their assortment. The *Price Conscious Consumers* attach little value to any of the attributes considered in our purchase experiment but price. This group will switch products, in general choosing the cheapest alterative. According to Jing and Wen [172] and Koçaş and Bohlmann [173], retailers can win this group of consumers by providing low-price alternatives (e.g., conventional sweet red pepper) or by providing price discounts (e.g., potentially for larger size packages). Finally, for the *Process Quality Supporters*, all process attributes are not only of relatively high but also of about equal importance in consumers’ purchase decisions while price seems to be of minor relevance. The low opt-out ratio of 1.7%, in addition, signals that this group can be retained if retailers offer red sweet peppers that fulfill at least one of the desired attribute levels. This, in fact, does not require a further differentiation beyond the one already suggested for the *Japan Lovers* and *Domestic Supporters*. However, large retailers that have the possibility of further differentiating their assortment can extract high price premia by offering organic red sweet peppers and/or red sweet peppers with a CRT approved in Taiwan. Along the same lines, smaller retailers may be able to run a successful niche strategy by focusing on the needs of this smaller segment.

The recommendation so far takes class membership as a given. Our findings, however, show that while sociodemographic factors provide little power to explain class membership (compare Models 1 and 2 in Table 10), a result in line with previous research [17,163,174], attitudinal and trust perceptions significantly influence class membership, explaining 23% of the models’ variance. Thus, assuming labeling to be sufficient for consumers to consider process attributes in their purchase decisions will likely prove wrong. For labeling to receive credence, it seems essential to implement effective control authorities and secure a high level of transparency to (re)gain and maintain consumers’ trust. Furthermore, our findings suggest a lack of knowledge regarding what organic implies, at least for the segment of *Price Conscious Consumers* (see discussion above). Accordingly, action is needed by private and public agencies to raise consumers’ knowledge regarding the standards behind organic products.

Last, some potential limitations of this study must be acknowledged. First, in this study, we focused on one product, fresh red sweet pepper, in one country. Whether our findings are transferrable to other products part of Taiwanese people’s daily diet should be addressed in further research. Also, a comparison of results across countries would be desirable. In particular, it would be interesting to carry out the same kind of study in China and/or Japan, and thus in competing markets, and investigate whether segments characterized by similar preferences and characteristics across locations exist. Respective insights could be valuable for producers in their decision to export or concentrate on local markets. Second, it has to be noted that the analysis is based on a hypothetical choice experiment. Thus, the results might suffer from social desirability bias. Third, to avoid consumer fatigue, we decided for four product attributes of red sweet pepper based on a pre-study. However, it must be considered that the selection of attributes and attribute levels may impact DCE outcomes. Along the same line, we limited the number of BWS statements to 25 as to cover all different dimensions of trust, with respect to all relevant characteristics of the products, would have resulted in more than 70 BWS statements, which would have been unmanageable. Nevertheless, there could be arguments for a different choice of statements than those selected that might have had an impact on the results. More specifically, we suggest for future studies to give more attention to capturing consumer animosity as a means to deepen the understanding about consumers’ attitude and perception, especially in the case of a negative COO image. Furthermore, in the questionnaire, participants were always requested to first answer the DCE questions and then the once of the BWS. We decided on this sequence to prevent consumers from knowing, while conducting the DCE, that the focus of the study was on trust. However, due to this order, we cannot rule out that the DCE had some influence on respondents’ answers in the BWS task.

Moreover, as we combined in our multinomial logit model the findings from a DCE latent class analysis (class membership entered as the dependent variable) and the outcome of the BWS Bayesian estimation (trust components entered as independent variables), we cannot rule out the problem of endogeneity. As we, however, lack an adequate instrumental variable, we cannot control for the endogenous issue. For future research, we suggest controlling for endogeneity and considering this issue by inclusion of an adequate covariate in the design of the survey. Finally, a simultaneous estimation of the DCE and BWS data would have been desirable. However, currently available software is incapable of estimating two large choice datasets (per individual 24 DCE rows and 100 BWS rows) concurrently in a single model. Therefore, such an analysis will only be possible in the future with the development of more sophisticated software.

## 5. Conclusions

Our study revealed that an analysis of what Taiwanese consumers value in their purchase of red sweet peppers falls short if it does not account for market heterogeneity. This holds as consumers (consumer segments) differ in the weights they assign to different product and process attributes. The findings of this paper, in addition, strongly support the argument that consumers’ attitudinal and trust perceptions are important determinants of class membership. These insights help to understand what actually drives class membership and to derive targeted marketing and policy strategies. From a methodological point of view, our paper is the first to combine a latent class model for discrete choices with BWS. The latter allowed us to capture consumers’ attitudinal and trust perceptions in a discriminant manner. Our results show that combining those methods enriches insights gained from latent class choice analysis.

## Figures and Tables

**Table 1:** Attributes and levels used in the Discrete Choice Experiment (DCE).

Attributes	Levels
Country of origin	Taiwan Japan China
Production method	Organic Conventional
Chemical residue testing information	Chemical residue test approved in the production country Chemical residue test approved in Taiwan No chemical residue test information provided
Price	NT 65 NT 85 NT 105 NT 125

In July 2014, 1 US Dollar = 29.98 New Taiwanese (NT) Dollars. 1 Taiwanese catty = 600 g.

**Table 2:** Nine dimensions of trust constructs with 25 Best-Worst Scaling (BWS) items.

Trust Constructs	Items Used in BWS Experiment	No. of Items	References
**1.** **Trust belief** in organic products from different COO	Taiwanese/Chinese/Japanese organic sweet peppers are trustworthy.	3	Adapted from the studies of [65,68,132].
2. **(Dis)trust belief** in the superior nutritional value of organic food	I feel sure that organic sweet peppers contain higher vitamin C and anti-cancer substances than conventional ones.	2	Adapted from the studies of [65,132].
	I feel sure that organic sweet peppers contain the same vitamin C and anti-cancer substances as conventional ones.		
**3.** **(Dis)Trust belief** in the environmental benefit of organic food	With purchasing organic sweet peppers, I help preserving the environment and natural resources.	2	Adapted from the studies of [65,132].
	There are no differences between buying organic sweet peppers or conventional ones with respect to preserving the environment and natural resources.		
**4.** **Trust belief** in the monetary value of organic food from different COO	Taiwanese/Chinese/Japanese organic sweet peppers have good value for money.	3	Adapted from the studies of [65,68].
5. **Trust intention** in purchasing products produced in different COO depending on chemical residue test information	It is more likely that I buy Taiwanese/Chinese/Japanese sweet peppers if information on chemical residue testing is provided.	3	Adapted from the studies of [65,68].
6. **Trust intention** in purchasing products produced in different COO depending on a price discount	It is more likely that I buy Taiwanese/Chinese/Japanese sweet peppers if it is on special offer.	3	Adapted from the studies of [65,68].
7. **Institutional-based trust** in governments of different countries	I feel assured that the Taiwanese/Chinese/Japanese institutions do a good job in adequately protecting consumers.	3	Adapted from the studies of [65].
8. **General trusting stance** regarding products produced from different COO	I generally like to consume conventional sweet peppers produced in Taiwan/China/Japan.	3	Adapted from the studies of [133,134].
9. **General trusting stance** in organic products from different COO	I generally like to consume organic sweet peppers produced in Taiwan/China/Japan.	3	

**Table 3:** Demographical statistics of the sample.

	Respondents		Taiwanese Population ^a^
Number of Respondents	459		
	Freq.	(%)	(%)
***Gender***			
Male	128	27.9	49.9
Female	331	72.1	50.1
***Responsibility for household food shopping***			
Fully	220	47.9	
Partly	239	52.1	
***Age***			
Up to 29	68	14.8	34.2
30–49	311	67.7	32.5
50 and over	78	17.0	33.4
Missing ^¶^	2	0.4	
***Marital status***			
Single	147	32.0	34.67
Married	284	61.9	51.12
Other (widowed/divorced)	23	5.0	14.21
Missing	5	1.1	
***Having children (<18 years old) in a household** (dummy coded: 1 = Yes; 0 = No)*	208	45.3	
***Education***			
Up to senior high school (12 years)	95	20.7	58.2
College (14 years)	119	25.9	11.4
University	233	50.8	30.4
Missing	12	2.6	
***Avg. monthly net income of the household***			
Up to NT 60,000	179	39.0	
NT 60,001–120,000	152	33.1	
NT 120,001 and over	56	12.2	
Missing	72	15.7	

^a^ Source: Ministry of the Interior, R.O.C. https://www.moi.gov.tw/; National Statistics, R. O. C. https://www.stat.gov.tw/. ^¶^ Two female participants did not give information about their age. As both already obtained a university degree, it seems reasonable to assume that they were above 18 years old, and thus eligible to participate in the survey. We therefore included their data in the analysis.

**Table 4:** Criteria for selecting the optimal number of classes.

Participants		459			
Null Log-Likelihood		−3817.85			
Groups	Log-Likelihood	Pct. Cert.	CAIC	Chi-Square	BIC
**2**	−2691.81	29.49	5517.43	2252.09	5502.43
**3**	−2557.13	33.02	5319.43	2521.46	5296.43
**4**	−2454.62	35.71	5185.87	2726.48	5154.78
**5**	−2397.36	37.21	5142.64	2840.99	5103.64
**6**	−2358.04	38.24	5135.36	2919.63	5088.36

**Table 5:** Latent class analysis of DCE data.

Null log-likelihood	−3817.85							
Restricted log-likelihood	−2454.62							
Pct. Cert.	35.71							
Consistent Akaike Info Criterion	5185.87							
Chi-Square	2726.38							
Bayesian Information Criterion	5154.87							
Segmentation	***1. Japan Lovers***		***2. Domestic Supporters***		***3. Price Conscious Consumers***		***4. Process Quality Supporters***	
Segment size (*N* = 459)	31.3%		26.1%		21.8%		20.8%	
	**Att. Imprt.**	**Rescaled Util. (S.E.)**	**Att. Imprt.**	**Rescaled Util. (S.E.)**	**Att. Imprt.**	**Rescaled Util. (S.E.)**	**Att. Imprt.**	**Rescaled Util. (S.E.)**
**COO**	59.83		71.66		27.36		32.26	
Taiwan		64.38 (0.22) ***		141.18 (0.21) ***		28.98 (0.18) ***		54.18 (0.18) ***
Japan		87.46 (0.11) ***		4.25 (0.13)		40.22 (0.10) ***		20.67 (0.10) ***
China		−151.85 (0.30) ***		−145.44 (0.29) ***		−69.20 (0.23) ***		−74.85 (0.23) ***
**Production methods**	6.83		5.23		3.36		32.45	
Organic		13.66 (0.06) ***		10.46 (0.07) ***		6.73 (0.07) *		64.90 (0.07) ***
Conventional		−13.66 (0.06) ***		−10.46 (0.07) ***		−6.73 (0.07) *		−64.90 (0.07) ***
**CRT**	8.85		6.90		15.33		27.98	
CRT appr. in prod. country		14.47 (0.08) ***		−1.39 (0.10)		−23.50 (0.09) ***		5.58 (0.09)
CRT appr.in TW		6.46 (0.08) **		14.50 (0.10) ***		37.84 (0.09) ***		53.16 (0.09) ***
No CRT		−20.94 (0.10) ***		−13.11 (0.12) **		−14.34 (0.12) **		−58.74 (0.12) ***
**Price**	24.49	−32.65 (0.13) ***	16.21	−21.61 (0.13) ***	53.95	−71.93 (0.10) ***	7.32	−9.75 (0.10) *
**No Choice**		−55.30 (0.19) ***		−26.87 (0.17) ***		−320.63 (0.37) ***		−156.05 (0.37) ***
**Share of No-Choice option**	8.69%		13.87%		0.17%		1.24%	

***, **, * Statistical significant level at 1%, 5%, and 10%. Att. Imprt. = Attribute importance. Rescaled part-worth utilities are zero-centered and normalized measures.

**Table 6:** Willingness to pay of different consumer segments.

Segmentation	*1. Japan Lovers*		*2. Domestic Supporters*		*3. Price Conscious Consumers*		*4. Process Quality Supporters*	
Segment Sizes (*N* = 459)	31.3%		26.1%		21.8%		20.8%	
(NT/600 g)	WTP	[95% C.I. Lower, Upper ^§^]	WTP	[95% C.I. Lower, Upper]	WTP	[95% C.I. Lower, Upper]	WTP	[95% C.I. Lower, Upper]
**COO**								
Taiwan	3.94	[3.16, 4.73]	13.07	[9.45, 16.68]	0.81	[0.44, 1.17]	11.11	[4.11, 18.11]
Japan	5.36	[4.46, 6.15]	0.39	[−0.28, 1.06]	1.12	[0.96, 1.27]	4.24	[2.35, 6.12]
China	−9.30	[−10.83, −7.77]	−13.46	[−17.05, −9.86]	−1.92	[−2.42, −1.43]	−15.35	[−24.09, −6.61]
**Production methods**								
Organic	0.84	[0.64, 1.04]	0.97	[0.51, 1.42]	0.19	[0.05, 0.32]	13.31	[4.27, 22.35]
Conventional	−0.84	[1.04, −0.64]	−0.97	[−1.42, −0.51]	−0.19	[−0.32, −0.05]	−13.31	[−22.35, −4.27]
**CRT**								
CRT appr. in prod. country	0.89	[0.65, 1.12]	−0.13	[−0.66, 0.41]	−0.65	[−0.73, −0.58]	1.14	[−0.28, 2.57]
CRT appr. in TW	0.40	[0.21, 0.58]	1.34	[0.69, 1.99]	1.05	[0.85, 1.25]	10.90	[3.92, 17.88]
No CRT	−1.28	[−1.59, −0.97]	−1.21	[−1.95, −0.48]	−0.40	[−0.60, −0.19]	−12.05	[−20.13, −3.96]

^§^ Upper and lower limits from the confidence intervals of WTP values, calculated with the delta method (Hole, 2007) at the 95% confidence level.

**Table 7:** Parallel analysis performance for component retention decisions across 25 BWS statements.

Component	Adjusted Eigenvalue	Unadjusted Eigenvalue	*Estimated Bias*
1	6.33	6.79	*0.46*
2	6.17	6.53	*0.36*
3	3.03	3.34	*0.31*
4	1.25	1.53	*0.28*
5	1.06	1.31	*0.25*

**Table 8:** Principal component analysis with oblimin rotation of 25 BWS items.

BWS Statement	Comp. 1 Trust in Japan	Comp. 2 Trust in Taiwan and Organics	Comp. 3 Trust in Chinese Products	Comp. 4 No Trust in Organics	Comp. 5 Trust in Chinese Organic Products
9. I generally like to consume organic sweet peppers produced in Japan.	0.890				
12. Japanese organic sweet peppers are trustworthy.	0.863				
4. I generally like to consume conventional sweet peppers produced in Taiwan.	−0.817				
20. It is more likely that I buy Taiwanese sweet peppers if it is on special offer.	−0.794				
25. Japanese organic sweet peppers have good value for money.	0.700				
10. Taiwanese organic sweet peppers are trustworthy.		0.866			
22. It is more likely that I buy Japanese sweet peppers if it is on special offer.		−0.837			
7. I generally like to consume organic sweet peppers produced in Taiwan.		0.818			
15. With purchasing organic sweet peppers, I help preserving the environment and natural resources.		0.785			
6. I generally like to consume conventional sweet peppers produced in Japan.		−0.758			
1. I feel assured that the Taiwanese institutions do a good job in adequately protecting consumers.		0.748			
19. It is more likely that I buy Japanese sweet peppers if information on chemical residue testing is provided.		−0.704			
13. I feel sure that organic sweet peppers contain higher vitamin C and anti-cancer substances than conventional ones.		0.679			
23. Taiwanese organic sweet peppers have good value for money.		0.631			
3 I feel assured that the Japanese institutions do a good job in adequately protecting consumers.		−0.517			
18. It is more likely that I buy Chinese sweet peppers if information on chemical residue testing is provided.			0.906		
21. It is more likely that I buy Chinese sweet peppers if it is on special offer.			0.749		
5. I generally like to consume conventional sweet peppers produced in China.			0.615		
14. I feel sure that organic sweet peppers contain the same vitamin C and anti-cancer substances as conventional ones.				−0.816	
16. There are no differences between buying organic sweet peppers or conventional ones with respect to preserving the environment and natural resources.				−0.777	
8. I generally like to consume organic sweet peppers produced in China.					0.930
24. Chinese organic sweet peppers have good value for money.					0.928
11. Chinese organic sweet peppers are trustworthy.					0.903
17. It is more likely that I buy Taiwanese sweet peppers if information on chemical residue testing is provided.					−0.828
2. I feel assured that the Chinese institutions do a good job in adequately protecting consumers.					0.454

**Table 9:** Total variance explained ^§^.

Component	Initial Eigenvalues		
	Total	Percentage of Variance	Cumulative Percentage
1	6.79	27.17	27.17
2	6.53	26.12	53.29
3	3.34	13.38	66.67
4	1.53	6.12	72.79
5	1.31	5.24	78.03
6	0.94	3.75	81.78
7	0.81	3.24	85.02
8	0.67	2.68	87.70
9	0.52	2.06	89.76
10	0.40	1.60	91.36
11	0.39	1.54	92.90
12	0.32	1.27	94.17
13	0.26	1.04	95.21
14	0.23	0.90	96.11
15	0.18	0.73	96.84
16	0.15	0.61	97.45
17	0.12	0.49	97.94
18	0.11	0.43	98.37
19	0.09	0.38	98.75
20	0.09	0.36	99.10
21	0.08	0.32	99.42
22	0.06	0.24	99.66
23	0.05	0.19	99.85
24	0.04	0.15	100.00
25	0.00	0.00	100.00

Extraction method: PCA. ^§^ The first five rows present the eigenvalues for the BWS individual level scores and percentage of variance for the five components.

**Table 10:** Multinomial logit models: DCE latent class membership regressed on five trust components (Model 1) and on five trust components plus sociodemographics (Model 2).

(*N* = 459)	Model 1		Model 2	
Log-likelihood of null model	−629.93		−629.93	
Log-likelihood of restricted model	−484.92		−469.61	
LR test Chi-square (33)	196.05		223.32	
Prob > Chi-square	0.00		0.00	
Pseudo R-squares	0.23		0.25	
DCE four segments	**Coef.**	**Robust Std. Err.**	**Coef.**	**Robust Std. Err.**
***Japan Lovers***	***Reference group***			
***Domestics Supporters***				
Trust in Japan	−0.84 ***	0.17	−0.81 ***	0.17
Trust in Taiwan & organic	1.29 ***	0.18	1.33 ***	0.19
Trust in Chinese products	−0.28	0.42	−0.10	0.45
No trust in organic	0.09	0.18	0.04	0.18
Trust in Chinese organic prod.	−0.10	0.29	−0.14	0.29
Full_HHShopResp			0.63 *	0.33
Female			−0.16	0.34
Age_below40			0.13	0.32
Have_Kids			−0.48	0.30
Edu_aboveCollege			−0.67 *	0.36
HHincome_above90k			−0.04	0.34
Constant	−0.42 *	0.22	0.12	0.52
***Price Conscious Consumers***				
Trust in Japan	−0.89 ***	0.18	−0.86 ***	0.19
Trust in Taiwan & organic	−0.03	0.17	0.09	0.17
Trust in Chinese products	1.19 ***	0.41	1.26 ***	0.49
No trust in organic	0.07	0.19	−0.01	0.20
Trust in Chinese organic prod.	0.52 *	0.29	0.49 *	0.30
Full_HHShopResp			−0.01	0.34
Female			−0.63 *	0.34
Age_below40			−0.48	0.35
Have_Kids			−0.79 **	0.34
Edu_aboveCollege			−0.90 **	0.44
HHincome_above90k			−0.16	0.35
Constant	−0.26	0.29	1.47 ***	0.59
***Process Quality Supporters***				
Trust in Japan	−0.36 **	0.17	−0.34 **	0.17
Trust in Taiwan & organic	0.80 ***	0.17	0.86 ***	0.18
Trust in Chinese products	0.67 *	0.40	0.75	0.47
No trust in organic	0.23	0.20	0.18	0.20
Trust in Chinese organic prod.	0.93 ***	0.23	0.90 ***	0.23
Full_HHShopResp			0.25	0.33
Female			−0.13	0.36
Age_below40			−0.48	0.31
Have_Kids			−0.15	0.31
Edu_aboveCollege			−0.63	0.42
HHincome_above90k			−0.15	0.37
Constant	−0.26	0.21	0.55	0.58

***, **, * Statistical significant level at 1%, 5%, and 10%.

## Appendix A. BWS Design Matrices

**Table:**

Number of statements = 25	
Number of statements per choice set = 5	
Number of sets per respondent: 10	
Number of blocks: 30	
**One Way Frequencies:**	
Statement	Frequencies Used
1	60
2	60
3	60
4	60
5	60
6	60
7	60
8	60
9	60
10	60
11	60
12	60
13	60
14	60
15	60
16	60
17	60
18	60
19	60
20	60
21	60
22	60
23	60
24	60
25	60
Mean =	60
S.D. =	0

**Table:**

Positional Frequencies:					
	Position in the BWS choice set				
Statement	1	2	3	4	5
1	12	12	12	12	12
2	11	13	12	12	12
3	12	13	12	12	11
4	12	11	13	12	12
5	12	12	12	13	11
6	12	13	11	12	12
7	12	12	12	13	11
8	12	12	12	11	13
9	12	11	12	12	13
10	12	12	12	12	12
11	12	12	12	12	12
12	12	13	12	11	12
13	12	12	12	12	12
14	12	12	12	12	12
15	12	11	12	13	12
16	12	12	12	12	12
17	12	12	12	12	12
18	11	12	12	12	13
19	12	12	12	12	12
20	13	12	12	11	12
21	12	12	13	11	12
22	13	12	11	12	12
23	12	11	12	13	12
24	12	12	12	11	13
25	12	12	12	13	11
Mean =	12				
S.D. =	0.522				

**Table:**

Two Way Frequencies:																									
Statement	1	2	3	4	5	6	7	8	9	10	11	12	13	14	15	16	17	18	19	20	21	22	23	24	25
1	60	10	11	10	10	10	10	10	10	10	10	11	9	11	10	10	10	10	10	10	9	10	9	10	10
2	10	60	10	10	10	10	10	10	10	10	10	10	9	10	10	11	10	9	10	10	11	10	10	10	10
3	11	10	60	10	10	9	10	10	10	9	10	10	11	10	10	10	10	10	11	9	10	10	10	10	10
4	10	10	10	60	10	10	9	10	10	10	9	10	10	10	10	10	11	11	10	10	10	10	10	10	10
5	10	10	10	10	60	10	10	10	10	10	10	10	10	10	10	10	10	10	10	9	10	11	10	10	10
6	10	10	9	10	10	60	10	11	10	10	10	10	10	10	9	10	10	10	11	10	10	10	9	10	11
7	10	10	10	9	10	10	60	10	10	11	11	10	10	11	9	10	9	10	10	10	10	10	10	10	10
8	10	10	10	10	10	11	10	60	10	9	10	10	11	10	10	10	10	10	11	10	9	10	10	10	9
9	10	10	10	10	10	10	10	10	60	10	10	10	11	9	11	9	10	11	9	10	10	10	10	10	10
10	10	10	9	10	10	10	11	9	10	60	10	10	10	10	11	9	11	10	10	10	10	10	10	10	10
11	10	10	10	9	10	10	11	10	10	10	60	9	11	9	11	11	10	10	10	10	10	10	10	9	10
12	11	10	10	10	10	10	10	10	10	10	9	60	9	11	11	11	9	9	10	10	10	10	10	10	10
13	9	9	11	10	10	10	10	11	11	10	11	9	60	10	10	10	10	10	9	10	10	10	10	10	10
14	11	10	10	10	10	10	11	10	9	10	9	11	10	60	10	9	9	10	10	10	10	10	11	10	10
15	10	10	10	10	10	9	9	10	11	11	11	11	10	10	60	10	10	10	9	11	10	9	10	10	9
16	10	11	10	10	10	10	10	10	9	9	11	11	10	9	10	60	10	10	10	10	10	10	10	10	10
17	10	10	10	11	10	10	9	10	10	11	10	9	10	9	10	10	60	10	10	10	10	10	10	10	11
18	10	9	10	11	10	10	10	10	11	10	10	9	10	10	10	10	10	60	9	10	10	10	10	11	10
19	10	10	11	10	10	11	10	11	9	10	10	10	9	10	9	10	10	9	60	9	11	11	10	10	10
20	10	10	9	10	9	10	10	10	10	10	10	10	10	10	11	10	10	10	9	60	11	10	11	10	10
21	9	11	10	10	10	10	10	9	10	10	10	10	10	10	10	10	10	10	11	11	60	9	10	10	10
22	10	10	10	10	11	10	10	10	10	10	10	10	10	10	9	10	10	10	11	10	9	60	10	10	10
23	9	10	10	10	10	9	10	10	10	10	10	10	10	11	10	10	10	10	10	11	10	10	60	10	10
24	10	10	10	10	10	10	10	10	10	10	9	10	10	10	10	10	10	11	10	10	10	10	10	60	10
25	10	10	10	10	10	11	10	9	10	10	10	10	10	10	9	10	11	10	10	10	10	10	10	10	60
Mean =		10																							
S.D. =		0.497																							

## Appendix B. Results of the Hierarchical Bayesian Mixed Logit Model for BWS Data

**Table:**

Total respondents	459		
Total Best Choices	4590		
Total Worst Choices	4590		
Root Likelihood statistics (model fit)	0.58		
**BWS Statements**	**Rank ^a^**	**Avg. Imprt. Scores ^b^**	**[95% C.I. Lower, Upper]**
1. I feel assured that the Taiwanese institutions do a good job in adequately protecting consumers.	12	4.80	[4.50–5.10]
2. I feel assured that the Chinese institutions do a good job in adequately protecting consumers.	24	0.08	[0.06–0.11]
3. I feel assured that the Japanese institutions do a good job in adequately protecting consumers.	10	5.43	[5.18–5.69]
4. I generally like to consume conventional red sweet peppers produced in Taiwan	14	4.26	[4.01–4.51]
5. I generally like to consume conventional red sweet peppers produced in China.	25	0.05	[0.03–0.07]
6. I generally like to consume conventional red sweet peppers produced in Japan.	18	1.98	[1.84–2.11]
7. I generally like to consume organic red sweet peppers produced in Taiwan.	4	7.30	[7.09–7.52]
8. I generally like to consume organic red sweet peppers produced in China.	22	0.33	[0.24–0.42]
9. I generally like to consume organic red sweet peppers produced in Japan.	13	4.68	[4.38–4.98]
10. Taiwanese organic red sweet peppers are trustworthy.	2	7.73	[7.52–7.93]
11. Chinese organic red sweet peppers are trustworthy.	21	0.49	[0.37–0.61]
12. Japanese organic red sweet peppers are trustworthy.	7	5.96	[5.67–6.24]
13. I feel sure that organic red sweet peppers contain higher vitamin C and anti-cancer substances than conventional ones.	16	3.24	[2.98–3.51]
14. I feel sure that organic red sweet peppers contain the same vitamin C and anti-cancer substances as conventional ones.	15	3.53	[3.23–3.83]
15. With purchasing organic red sweet peppers I help preserve the environment and natural resources.	11	5.29	[5.01–5.57]
16. There are no differences between buying organic red sweet peppers or conventional ones with respect to preserving the environment and natural resources.	17	2.32	[2.09–2.54]
17. It is more likely that I buy Taiwanese red sweet peppers if information on chemical residue testing is provided.	1	8.42	[8.21–8.62]
18. It is more likely that I buy Chinese red sweet peppers if information on chemical residue testing is provided.	19	0.75	[0–0.92]
19. It is more likely that I buy Japanese red sweet peppers if information on chemical residue testing is provided.	5	7.27	[7–7.48]
20. It is more likely that I buy Taiwanese red sweet peppers if they are on special offer.	6	6.52	[6–6.84]
21. It is more likely that I buy Chinese red sweet peppers if they are on special offer.	23	0.29	[0–0.39]
22. It is more likely that I buy Japanese red sweet peppers if they are on special offer.	8	5.78	[5–6.08]
23. Taiwanese organic red sweet peppers have good value for money.	3	7.39	[7–7.61]
24. Chinese organic red sweet peppers have good value for money.	20	0.50	[0.37–0.64]
25. Japanese organic red sweet peppers have good value for money.	9	5.61	[5–5.92]

^a^ Rank indicates the rank position among the 25 statements based on the importance score. ^b^ Avg. imprt. Scores = Average importance scores.
